# Antimicrobial Resistance Profiles, Virulence Genes, and Genetic Diversity of Thermophilic *Campylobacter* Species Isolated From a Layer Poultry Farm in Korea

**DOI:** 10.3389/fmicb.2021.622275

**Published:** 2021-03-29

**Authors:** Noel Gahamanyi, Dae-Geun Song, Kye-Yoon Yoon, Leonard E. G. Mboera, Mecky I. Matee, Dieudonné Mutangana, Raghavendra G. Amachawadi, Erick V. G. Komba, Cheol-Ho Pan

**Affiliations:** ^1^Natural Product Informatics Research Center, KIST Gangneung Institute of Natural Products, Gangneung, South Korea; ^2^SACIDS Foundation for One Health, College of Veterinary Medicine and Biomedical Sciences, Sokoine University of Agriculture, Morogoro, Tanzania; ^3^School of Medicine, Muhimbili University of Health and Allied Sciences, Dar es Salaam, Tanzania; ^4^College of Science and Technology, University of Rwanda, Kigali, Rwanda; ^5^Department of Clinical Sciences, College of Veterinary Medicine, Kansas State University, Manhattan, KS, United States; ^6^Division of Bio-Medical Science and Technology, KIST School, Korea University of Science and Technology, Seoul, South Korea

**Keywords:** *Campylobacter*, quinolones, antimicrobial resistance, *fla*A RFLP, multilocus sequence typing, poultry, Korea

## Abstract

Thermophilic *Campylobacter* species are among the major etiologies of bacterial enteritis globally. This study aimed at assessing the antimicrobial resistance (AMR) profiles, virulence genes, and genetic diversity of thermophilic *Campylobacter* species isolated from a layer poultry farm in South Korea. One hundred fifty-three chicken feces were collected from two layer poultry farms in Gangneung, South Korea. The *Campylobacter* species were isolated by cultural techniques, while PCR and sequencing were used for species confirmation. Antimicrobial susceptibility testing for six antimicrobials [ciprofloxacin (CIP), nalidixic acid (NAL), sitafloxacin (SIT), erythromycin (ERY), tetracycline (TET), and gentamicin (GEN)] was carried out by broth microdilution. Three AMR and nine virulence genes were screened by PCR. Genotyping was performed by *fla*A-restriction fragment length polymorphism (RFLP) and multilocus sequence typing (MLST). Of the 153 samples, *Campylobacter* spp. were detected in 55 (35.9%), with *Campylobacter jejuni* and *Campylobacter coli* being 49 (89.1%) and six (10.9%), respectively. High-level resistance was observed for CIP (100%), NAL (100%), and TET (*C. jejuni*, 93.9%; *C. coli*: 83.3%). No resistance was observed for SIT. The missense mutation (C257T) in *gyr*A gene was confirmed by sequencing, while the *tet*(O) gene was similar to known sequences in GenBank. The rate of multidrug-resistant (MDR) strains was 8.2%, and they all belonged to *C. jejuni*. All *Campylobacter* isolates possessed five virulence genes (*cdt*B, *cst*II, *fla*A, *cad*F, and *dna*J), but none possessed *ggt*, while the rates for other genes (*csr*A, *cia*B, and *pld*A) ranged between 33.3 and 95.9%. The *fla*A-RFLP yielded 26 *fla*A types (*C. jejuni*: 21 and *C. coli*: five), while the MLST showed 10 sequence types (STs) for *C. jejuni* and three STs for *C. coli*, with CC-607 (STs 3611) and CC-460 (ST-460) being predominant. Among the 10 STs of *C. jejuni*, three were newly assigned. The findings of this study highlight the increased resistance to quinolones and TET, the virulence potential, and the diverse genotypes among *Campylobacter* strains isolated from the layer poultry farm.

## Introduction

Globally, *Campylobacter* is the leading cause of bacterial gastroenteritis (Alaboudi et al., 2020). *Campylobacter jejuni* and *Campylobacter coli* are the species of clinical significance, being accountable for more than 95% of human campylobacteriosis (Moore et al., 2005; Fitzgerald, 2015). Globally, 96 million cases of diarrhea each year are due to *Campylobacter* (Havelaar et al., 2015). In Europe, *Campylobacter* was ranked as the second (next to *Salmonella*) etiological agent of outbreaks associated with water and food poisoning in 2018 (Klančnik et al., 2020). Contrary to European countries, reports on human campylobacteriosis in Asian countries including South Korea are limited, possibly due to low disease prevalence or the sporadic nature of infections (Kim et al., 2019; Wei et al., 2019).

Human campylobacteriosis requires antimicrobial therapy only in case of complications and in immuno-compromised people (Wieczorek and Osek, 2013; Sproston et al., 2018). Over the years, increasing rates of *Campylobacter* strains that are resistant to the drugs of choice [fluoroquinolones (FQs) and macrolides] and alternative therapies [gentamicin (GEN) and tetracycline (TET)] have been reported (Blaser and Engberg, 2008; Koolman et al., 2015), making antimicrobial resistant (AMR) *Campylobacter* strains a public health concern (Mourkas et al., 2019; Windiasti et al., 2019). The resistance to antimicrobials is partly due to their misuse in both human medicine and livestock production (Elisha et al., 2017; Sproston et al., 2018). For instance, different quinolone antibiotics have been extensively used in poultry raising, which led to an accelerated number of quinolone-resistant strains of *Campylobacter* from chicken and humans (Sproston et al., 2018).

In Korea, FQ use in livestock was banned since 2010, with a prediction to curb the increased resistance in the future (Ku et al., 2011). However, recently, FQ-resistant *Campylobacter* strains have been isolated from poultry and duck meat (Kim et al., 2019). Due to the increased resistance to quinolones throughout the world (Tang et al., 2017), erythromycin (ERY) has emerged as the recommended drug for treating human campylobacteriosis (Giannatale et al., 2019). Recently, sitafloxacin (SIT), a novel FQ drug, proved to be effective against various FQ-resistant pathogens including *Campylobacter* (Changkwanyeun et al., 2016; Chen et al., 2020), and could be a promising drug. The persistence of FQ resistance in *Campylobacter* strains could be linked to the continued use of ciprofloxacin (CIP) in human medicine, international trade, travel, and use of FQs in animal husbandry along with the circulation of resistant isolates among different reservoirs.

Poultry can carry *Campylobacter*, and chicken intestines are regarded as reservoirs for thermophilic *Campylobacter* species based on optimal conditions (high body temperature) favoring their growth (Sibanda et al., 2018). Previous reports have associated an increase in human campylobacteriosis cases with the increase in chicken consumption (Oh et al., 2017). Chickens’ ceca alone are usually colonized by *C. jejuni* to levels above 10^9^ colony-forming unit (CFU)/ml, posing a risk to humans (Humphrey et al., 2007, 2014). Furthermore, *Campylobacter* can stay in feces and litter for many days, and the use of these byproducts as fertilizers would aggravate the dissemination of the pathogens (Kassem et al., 2016). *Campylobacter* persistence in chicken farms and slaughterhouses is a hazard to the consumers because it is transmitted along the whole production chain up to the final product (Kim et al., 2019; Ramires et al., 2020). While the literature on broiler chicken is extensive, studies on the epidemiology of *Campylobacter* species from layer farms are limited (Kassem et al., 2016).

*Campylobacter* species are equipped with virulome which is used in attachment, establishment, invasion, and production of toxins, contributing to their increased occurrence and epidemiology compared to other enteric bacteria (Bolton, 2015; Otigbu et al., 2018). However, the mechanisms associated with *Campylobacter* pathogenicity are not fully understood (Nguyen et al., 2016). *C. jejuni* is known to cause Guillain–Barré syndrome, characterized by acute and progressive neuromuscular paralysis, mediated by sialyltransferases (*cst*II) (Koga et al., 2006; Humphrey et al., 2007; González-Hein et al., 2013). Sialic acid confers immune avoidance to *C. jejuni*, as a mutant lacking lipooligosaccharide sialic acid residues showed greater immunoreactivity and decreased serum resistance (Kreling et al., 2020). The CDT complex, another important factor in *Campylobacter*, codes for the cytolethal distending toxin with *cdt*B acting as the catalytic site, and in the nucleus, *cdt*B induces cell cycle arrest and leads to apoptosis of both immune and epithelial cells in the intestines (Jain et al., 2008). It has been reported that *C. jejuni cdt*B mutants had reduced extra-intestinal invasiveness (Yamasaki et al., 2006) and bowel disturbances (Pokkunuri et al., 2012). A study carried out in Poland showed that *Campylobacter* strains lacking *cdt*B and *cdt*C were non-cytotoxic, confirming their roles in toxin production (Wysok et al., 2020). The presence of *ggt* contributes to the colonization potential of *C. jejuni* in chicken and mice intestines (Barnes et al., 2007). The *fla*A gene contributes to *Campylobacter* pathogenesis as it is involved in colonization, motility, auto-agglutination, and biofilm formation (Guerry, 2007). Mutation experiments highlighted the role of *fla*A in chicken colonization (Bolton, 2015). *Campylobacter* species also possess other genes associated with adhesion (*cad*F and *pld*A), invasion (*cia*B), thermo-tolerance (*dna*J) (Pillay et al., 2020), and stress response (*csr*A) (Fields and Thompson, 2008). Studies have shown that *Campylobacter* strain mutants for *cad*F and *cia*B exhibited a reduced attachment and invasion of INT 407 cell line along with a decline of survival potential (Kreling et al., 2020; Ramires et al., 2020).

Molecular typing methods are important not only in distinguishing bacteria at the species and subspecies levels but also in source attribution of *Campylobacter* strains (Eberle and Kiess, 2012; Lydekaitiene and Kudirkiene, 2020). Although source attribution aiming at quantifying the contribution of different reservoirs, pathways, exposures, and risk factors to the burden of human illness is difficult (Wagenaar et al., 2013), it is estimated that 80% of human campylobacteriosis are attributed to *Campylobacter* of poultry origin (Wagenaar et al., 2013; Mulder et al., 2020). Multilocus sequence typing (MLST), based on seven housekeeping genes (HKGs), is the gold-standard method used for epidemiological surveillance (Harrington et al., 2003; Lydekaitiene and Kudirkiene, 2020). Data on MLST of *Campylobacter* strains in Asia are limited (Nguyen et al., 2016), but previous studies in Korea and Japan have shown the predominance of CC-460, CC-607, CC-21, and CC-45 in poultry and human isolates (Wei et al., 2014; Ozawa et al., 2016; Oh et al., 2017). MLST data are expected to give accurate phylogenic estimation, typing, and strain relatedness (Alaboudi et al., 2020). Whole-genome sequencing (WGS) might become the preferred typing method in the future, but still, there is a need for a consensus upon bioinformatics pipelines and tools for processing WGS data (Duarte et al., 2016b).

Considering the persistence of FQ-resistant *Campylobacter* strains even in the absence of antimicrobial use and the fact that SIT has a different structure compared to other FQs, we hypothesized that *Campylobacter* species from chicken are still resistant to both ciprofloxacin and nalidixic acid (NAL) but sensitive to SIT. Furthermore, we think that the same sequence types (STs) are circulating in poultry in Korea and the region. Based on the virulence potential of *Campylobacter* and the favorable environment offered by chicken, it is most probable that *Campylobacter* species of poultry origin are hypervirulent and could be of concern. The present study aimed at assessing the antimicrobial resistance profiles, virulence genes, and genetic diversity of thermophilic *Campylobacter* species isolated from a layer poultry farm in Korea.

## Materials and Methods

### Sample Collection

Fresh chicken fecal samples were purposively collected from two layer poultry farms located in Gangneung city, Republic of Korea in June 2020. The first farm uses an intensive poultry farming system with around 800 1-year-old chickens dispatched into battery cages inside a closed house. The second farm is a small one that is not for commercial purposes, where around 30 1-year-old chickens are enclosed in a cage subdivided into two blocks by a fence. A total of 133 (from the first farm) and 20 (from the second farm) pen floor fecal samples were collected using sterile swabs and transported to the laboratory under refrigeration (ice) within 1 h.

### *Campylobacter* Isolation and Antimicrobial Susceptibility Testing

Upon arrival at the laboratory, the feces were inoculated onto modified charcoal cefoperazone deoxycholate agar (mCCDA) (Oxoid Ltd., Basingstoke, Hampshire, England) containing the *Campylobacter* mCCDA-selective supplement, SR155E (Oxoid Ltd, Basingstoke, Hampshire, England). Incubation was done as previously described (Kurekci et al., 2012) at 37°C for 48 h under microaerophilic conditions generated by CampyGen^TM^ gas sachets (Oxoid, Basingstoke, England, United Kingdom). Typical colonies of *Campylobacter*, which are grayish, flat, moistened, and with a tendency to spread (Al-Edany et al., 2015), were sub-cultured on Mueller Hinton Agar supplemented with 5% defibrinated horse blood (MHS) and incubated at 37°C for 48 h under microaerophilic conditions (Kurekci et al., 2012). *Campylobacter* isolates were preserved at −80°C in Mueller Hinton broth containing 25% glycerol (v/v).

Antimicrobial susceptibility testing was performed by broth microdilution. The isolates were tested against quinolones, namely ciprofloxacin (CIP), NAL (0.25–512 μg/ml), SIT (0.03–16 μg/ml); macrolide, ERY (0.06–64 μg/ml); and aminoglycoside, GEN (0.06–64 μg/ml), and TET (0.125–1,024 μg/ml). Apart from SIT purchased from AdooQ BioScience (Irvine, CA, United States), the other antimicrobials were supplied by Sigma-Aldrich (St. Louis, MO, United States). CIP and ERY were dissolved in 0.1 N HCl and 70% ethanol, respectively. GEN and TET were dissolved in water, while NAL and SIT were dissolved in dimethyl sulfoxide (DMSO). Except for the antibiotics dissolved in DMSO, other solutions of antibiotics were filter-sterilized before being used.

Preserved *Campylobacter* isolates were inoculated onto MHS (Oxoid Ltd, Basingstoke, Hampshire, England) and incubated at 37°C for 48 h under microaerophilic conditions (Kurekci et al., 2012). A sub-culture was performed on the same media and the same conditions to get well-grown pure colonies free from glycerol. For antimicrobial susceptibility assays, suspensions corresponding to 0.5 McFarland standard (1.5 × 10^8^ CFU/ml) were prepared using normal saline, and the final concentration in a 96-well plate was 2–5 × 10^6^ CFU/ml. The minimal inhibitory concentration (MIC) was determined by checking the absorbance at *A*_600_ nm on a microplate reader (Synergy HT; BioTek Instruments Inc., Winooski, VT, United States) and confirmed by the addition of iodonitrotetrazolium chloride to 96-well plates as previously described (Klančnik et al., 2009). The MIC was designated as the lowest concentration of the antimicrobial leading to a significant decrease (>90%) in inoculum viability after 48 h as previously described with modification on incubation time (Burt, 2004). The minimal bactericidal concentration (MBC) was determined as previously described (Dholvitayakhun and Trachoo, 2012; Duarte et al., 2016a). The concentration at which no bacterial growth was noticed after 48 h of incubation was regarded as MBC. The MIC values were interpreted according to the standards of the European Committee for Antimicrobial Susceptibility Testing^1^, except for SIT which lacks international cutoff values. The MIC values were CIP ≤ 0.5 μg/ml, NAL ≤ 16 μg/ml, ERY ≤ 4 for *C. jejuni* and ≤8 μg/ml for *C. coli*, and GEN and TET ≤ 2 μg/ml. However, all *Campylobacter* strains were sensitive to SIT (MIC ≤ 2 μg/ml) according to the literature (Huang et al., 2015; Xu et al., 2018).

### DNA Extraction, PCR Confirmation of Species, and Detection of AMR and Virulence Genes

The extraction of genomic DNA from pure colonies was carried out by using the Qiagen QIAamp PowerFecal Kit (Qiagen, Hilden, Germany) as per the manufacturer’s instructions. Then, a multiplex PCR was conducted using genus-specific primers (C412F and C1228R), *cj0414* gene primers (C1 and C3) for *C. jejuni*, and *ask* gene primers (CC18F and CC519R) for *C. coli* (Yamazaki-Matsune et al., 2007). The primers were selected based on the specificity in identifying the genus and species of *Campylobacter* (Linton et al., 1997; Pajaniappan et al., 2008). The PCR mixture (25 μl) contained 12.5 μl of 2X Master Mix (Thermo Fisher Scientific, Seoul, South Korea), 1 μl of primer (10 μM), 1.5 μl of template DNA (20 μg/ml), and 7 μl of sterile deionized water. The cycling conditions were one cycle of 95°C for 5 min, 35 cycles each of 94°C for 30 s, 55°C for 45 s, and 72°C for 45 s, and a final extension at 72°C for 7 min using MiniAmp Plus Thermal Cycler (Applied Biosystems, MA, United States). The PCR products were held at 4°C before analysis.

**FIGURE 1:**
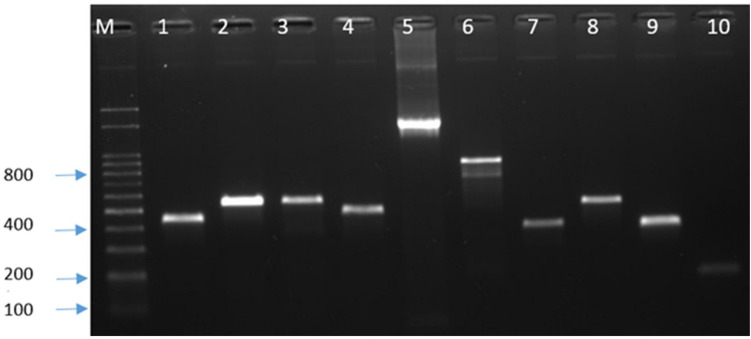
PCR detection of antimicrobial resistance and virulence genes. M, marker; 1, *gyr*A; 2, *tet*(O); 3, *cst*II; 4, *cdt* B; 5, *fla*A; 6, *csr*A; 7, *cad*F; 8, *cia*B; 9, *pld*A; and 10, *dna*J.

For the genes associated with antibiotic resistance [*tet*(O), *gyr*A, and *cme*B] and virulence (*cst*II, *cdt*B, *fla*A, *ggt*, *csr*A, *cad*F, *cia*B, *pld*A, and *dna*J), the PCR was performed using specific primers (Supplementary Table 1). After electrophoresis, bands of PCR products (Figure 1) were observed on a Dual UV Transilluminator (Core Bio System, Huntington Beach, CA, United States) under ultraviolet light. The bands of the amplification products were compared to the 100-bp DNA ladder (Dyne bio, Seongnam-si, Republic of Korea). The PCR products of antibiotic resistance genes were purified with AMPure XP beads (Beckman Coulter, Fullerton, CA, United States) and sequenced by the Sanger method at SolGent (Solutions for Genetic Technologies, Daejeon, Republic of Korea).

### *fla*A-Restriction Fragment Length Polymorphism

Genetic diversity was first analyzed by *fla*A-restriction fragment length polymorphism (RFLP) using 25-μl PCR reactions (Harrington et al., 2003; Wieczorek and Osek, 2008). The *fla*A amplicon (1.7 kb) was digested for 6 h at 37°C using HpyF3I restriction enzyme (Thermo Scientific, Waltham, MA, United States), and the fragments were separated using 2.5% agarose gel (Lonza Inc., Rockland, ME, United States) in Tris–acetate–EDTA buffer at 90 V for 90 min. The bands were photographed with iBright^TM^ CL1000 Imaging System (Thermo Fisher Scientific, Seoul, Republic of Korea). The dyne 100-bp and 1-kb DNA ladders (Dyne bio, Seongnam-si, Republic of Korea) were used as standards for molecular size determination.

### Multilocus Sequence Typing Analysis

Multilocus sequence typing was performed as previously described (Dingle et al., 2001; Giannatale et al., 2019) using primers available from the *Campylobacter* MLST website^2^. Briefly, the seven HKGs *asp*A (aspartase), *gln*A (glutamine synthetase), *glt*A (citrate synthase), *gly*A (serine hydroxymethyltransferase), *pgm* (phosphor glucomutase), *tkt* (transketolase), and *unc*A (ATP synthase) were PCR-amplified from genomic DNA. For *C. jejuni*, two rounds of PCR were performed (nested PCR), while for *C. coli* one set of primers was used. The PCR conditions were denaturation at 94°C for 5 min, 35 cycles of 94°C for 30 s, 60°C for 45 s, and 72°C for 45 s, and then a final extension at 72°C for 5 min. The purification of amplicons was performed by AMPure XP beads (Beckman Coulter, Fullerton, CA, United States) as per the manufacturer’s recommendations and sequenced by the Sanger method at SolGent (Solutions for Genetic Technologies, Daejeon, Republic of Korea).

### Data Analysis

GraphPad Prism 8.4.0 (GraphPad Software, La Jolla, CA, United States) was used to compute the descriptive statistics (detection rate, proportions, and frequencies of different attributes). The *fla*A restriction profiles were analyzed by pairwise comparisons and cluster analysis using the Dice correlation coefficient and the unweighted pair group method with arithmetic mean clustering algorithm in BIONUMERICS V8.0 (Applied Maths, Sint-Martens-Latem, Belgium). The optimization and position tolerance (1%) for band analysis and a cutoff of 100% were used. BioEdit software (version 7.2.6.1) was used to edit, align, and analyze the DNA chromatograms (Hall, 1999). A BLAST search was performed to compare consensus sequences [*gyr*A and *tet*(O)] with those from the GenBank database. Then, our sequences were submitted to get the corresponding accession numbers. Standard sensitive strains (L04566.1 and U63413.1) and resistant strains (KX982339.1 and MT176401.1) for *gyr*A were used for comparison. For the *gyr*A gene, the comparison was performed with Clustal Omega (Madeira et al., 2019). Amino acid sequences were deduced from the DNA sequences using the ExPASyTranslate tool (Gasteiger et al., 2003). Alleles, STs, and clonal complexes (CCs) were assigned by submitting the sequence data to the MLST database (see text footnote 2) (Maiden, 2006). A minimum spanning tree of *C. jejuni* and *C. coli* STs was created from MLST allelic profiles using BIONUMERICS 8.0 (Applied Maths NV, Saint-Martens-Latem, Belgium).

## Results

Out of 153 fecal samples, the detection rate of *Campylobacter* spp. was 35.9% (55), with *C. jejuni* and *C. coli* being 89.1% (49) and 10.9% (six), respectively. None of the 20 fecal samples from the second farm was positive for *Campylobacter*.

### Antimicrobial Susceptibility Testing

All *Campylobacter* isolates were screened for antimicrobial susceptibility to six antimicrobials, and they showed high-level resistance to CIP, NAL, and TET. Resistance to CIP and NAL was 100%, while resistance to TET was 93.9% for *C. jejuni* and 83.3% for *C. coli* (Table 1). Four (8.2%) *C. jejuni* strains were multidrug-resistant (MDR), but none of the *C. coli* was MDR. Of the four MDR isolates, two were resistant to CIP, NAL, TET, and ERY, while the other two were resistant to CIP, NAL, TET, and GEN. The presence of *tet*(O) and mutation in *gyr*A were confirmed by PCR (Figure 1), but all strains did not show bands for the multidrug efflux pump gene (*cme*B). Sequencing revealed the presence of a missense mutation (C257T) in the quinolone resistance determining region of *gyr*A gene, causing resistance to quinolones along with other silent mutations. There was 100% sensitivity to SIT, while 4.1% of the *C. jejuni* isolates were resistant to both ERY and GEN (Table 1). The MBC values were as follows: CIP, 64–256 μg/ml; NAL, 128–512 μg/ml; TET, 2–1,024 μg/ml; SIT, 0.25–1 μg/ml; ERY, 1–32 μg/ml; and GEN, 1–256 μg/ml.

**TABLE 1:** Antimicrobial resistance data for both *Campylobacter jejuni* and *Campylobacter coli* species.

Anti-microbial	Class	Species	Resistance (%)	Number of isolates at the indicated minimal inhibitory concentration (μg/ml)													
				0.06	0.13	0.25	0.5	1	2	4	8	16	32	64	128	256	512
		*C. jejuni*	100	0	0	0	0	0	0	0	0	1	36	10	2	0	0
CIP		*C. coli*	100	0	0	0	0	0	0	0	0	0	4	2	0	0	0
	Fluoroquinolone	*C. jejuni*	0	2	33	11	2	1	0	0	0	0	0	0	0	0	0
SIT		*C. coli*	0	0	0	5	1	0	0	0	0	0	0	0	0	0	0

		*C. jejuni*	100	0	0	0	0	0	0	0	0	0	1	25	17	6	0
NAL	Quinolone	*C. coli*	100	0	0	0	0	0	0	0	0	0	0	1	5	0	0

		*C. jejuni*	4.1	0	0	1	38	3	2	3	1	1	0	0	0	0	0
ERY	Macrolide	*C. coli*	0	0	0	0	1	0	4	1	0	0	0	0	0	0	0

	Aminoglycoside	*C. jejuni*	4.1	0	1	11	27	8	0	0	0	0	0	2	0	0	0
GEN		*C. coli*	0	0	0	2	1	3	0	0	0	0	0	0	0	0	0

	Tetracycline	*C. jejuni*	93.9	0	0	0	2	1	0	0	0	0	3	10	22	10	1
TET		*C. coli*	83.3	0	0	0	1	0	0	0	0	0	0	1	2	2	0

Upon submission of the *gyr*A and *tet*(O) sequences to the GenBank database, the following accession numbers have been assigned: MW067325–MW067370 (Table 2). The main mutation in *gyr*A gene is the missense mutation (C257T) associated with the codon change from ACA to ATA (*C. jejuni*) and ACT to ATT (*C. coli*) leading to *T86I* substitution. However, silent mutations were also found. It was noticed that TET-resistant strains possessed the *tet*(O) gene, which was confirmed by sequencing. The BLAST search showed similarity with known *tet*(O) gene sequences in GenBank.

**TABLE 2:** Accession numbers for DNA *gyr*A and *tet*(O) resistance genes.

Isolate	DNA *gyr*A accession number	*tet*(O) accession number
CJ5	MW067325	MW067326
CJ8	MW067327	MW067328
CJ17	MW067329	MW067350
CJ21	MW067330	MW067351
CJ28	MW067331	MW067352
CJ32	MW067332	MW067353
CJ37	MW067333	MW067354
CJ42	MW067334	MW067355
CJ46	MW067335	MW067356
CJ48	MW067336	MW067357
CJ50	MW067337	MW067358
CJ51	MW067338	MW067359
CJ52	MW067339	MW067360
CJ53	MW067340	MW067361
CJ54	MW067341	MW067362
CJ55	MW067342	MW067363
CJ56	MW067343	MW067364
CJ60	MW067344	MW067365
CJ71	MW067345	MW067366
CC13	MW067346	MW067367
CC45	MW067347	MW067368
CC47	MW067348	MW067369
CC2	MW067349	MW067370
CC1	MT947449	MT967270

*CJ, Campylobacter jejuni; CC, Campylobacter coli.*

### Virulence Genes

**FIGURE 2:**
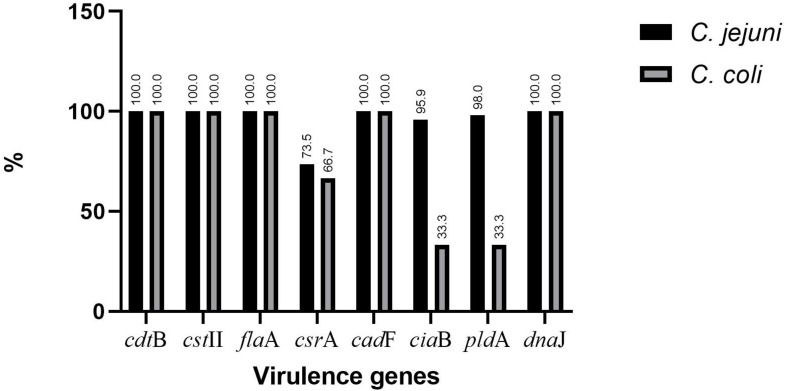
Distribution of virulence genes in *Campylobacter jejuni* and *Campylobacter coli*.

The presence of selected virulence genes (*cst*II, *cdt*B, *fla*A, *ggt*, *csr*A, *cad*F, *cia*B, *pld*A, and *dna*J) was checked by PCR (Figure 1). We found that all isolates possessed *cdt*B, cstII, *fla*A, *cad*F, and *dna*J, but none showed the presence of *ggt*. The percentages for *csr*A, *cia*B, and *pld*A were 73.5, 95.9, and 98% for *C. jejuni* and 66.7, 33.3, and 33.3% for *C. coli*, respectively (Figure 2).

### The *fla*A Polymorphism

**FIGURE 3:**
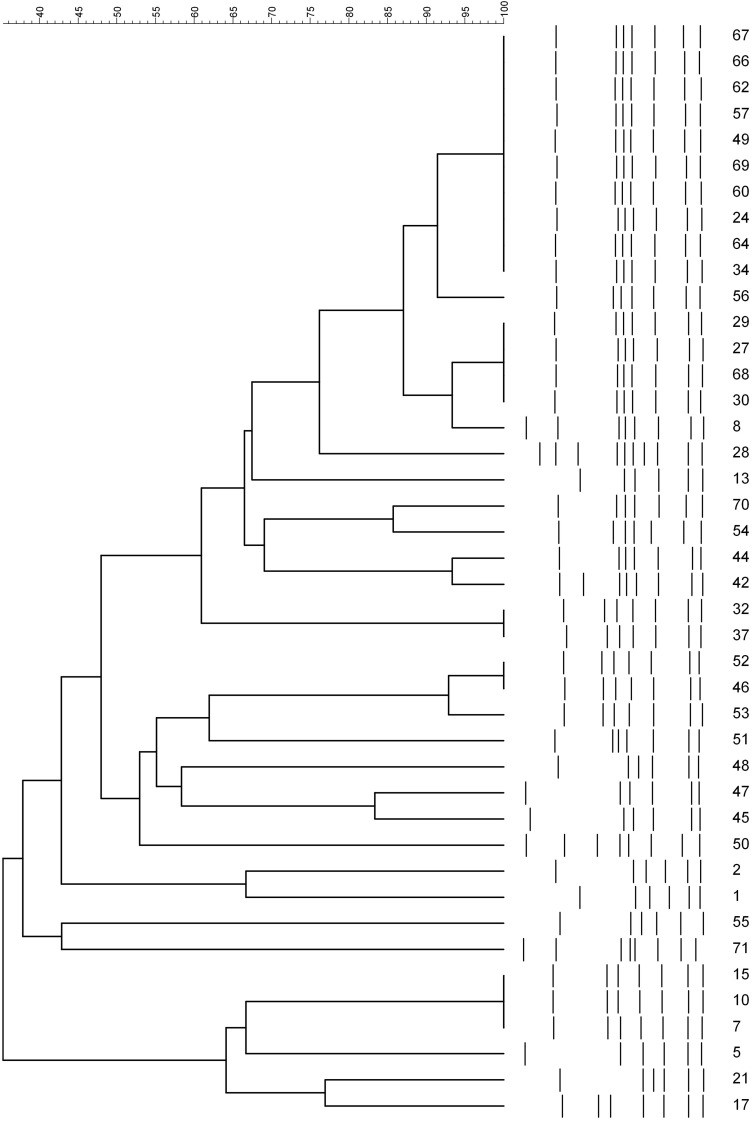
PCR–restriction fragment length polymorphism profiles of *fla*A gene digested with HpyF31 from strains of *Campylobacter* species. Numbers represent the laboratory code for isolates.

*Campylobacter jejuni* (*n* = 37) and *C. coli* (*n* = 5) were digested with HpyF3I, yielding six to 10 fragments of DNA. There were 26 *fla*-A types (*C. jejuni*: 21 and *C. coli*: five). For *C. jejuni*, there was a predominant cluster at the top of the dendrogram. For *C. coli*, isolate numbers 45 and 47 clustered together; the other isolates had different patterns (Figure 3).

### Multilocus Sequence Typing

Twenty-four isolates (*C. jejuni*: 19; *C. coli*: five), selected based on *fla*A RFLP profiles to maximize the diversity, were genotyped by MLST. *C. jejuni* isolates were matched with 10 STs grouped into five CCs. However, three *C. jejuni* isolates had new combinations of previously described alleles but could not be matched with any of the existing STs. Upon submission to the database (see text footnote 2) for ST assignment, the isolates CJ42 (id: 106369), CJ52 (id: 106370), and CJ71 (id: 106371) were assigned to ST-10645, ST-10647, and ST-10648, respectively. Of the 10 STs, ST-3611 was the main one with five isolates, followed by ST-460 with four isolates. The CCs with a higher number of isolates were CC-607 with nine isolates and CC-460 with five isolates. For *C. coli* (five isolates), three STs were found, with ST-5935 being the most prevalent (three isolates), and all the five isolates belonged to CC-1150 (Table 3).

**TABLE 3:** Distribution of sequence types and clonal complexes among *Campylobacter* strains from chicken (*n* = 24).

Species	Isolate ID	Sequence type	Total number	Clonal complex
*Campylobacter*	CJ5; CJ50	51	2	443
*jejuni*				
	CJ8; CJ51; CJ54; CJ60	460	4	460
	CJ56	10613	1	
	
	CJ17; CJ32; CJ37; CJ46; CJ53	3611	5	607
	CJ21; CJ48	607	2	
	CJ55	6238	1	
	CJ52	10647	1	
	
	CJ28	8994	1	52
	CJ42	10645	1	
	
	CJ71	10648	1	446
	
*Campylobacter*	CC1; CC13; CC45	5935	3	1150
*coli*	CC2	8164	1	
	CC47	1121	1	

**FIGURE 4:**
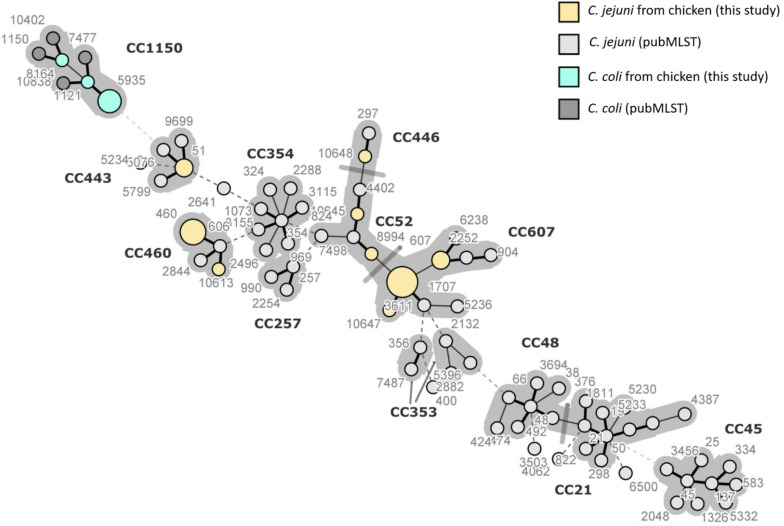
Minimum spanning tree of *Campylobacter jejuni* and *Campylobacter coli* sequence types (STs) created from multilocus sequence typing allelic profiles. Each ST is represented as a circle, with the size of the circle proportional to the number of isolates of that ST for the isolates obtained in this study (STs colored in yellow and green). The figure also includes STs commonly reported in Korea and elsewhere from poultry and humans which were retrieved from pubMLST. The branch length and thickness represent the allelic distance: a thick short line connects single-locus variants, a thin longer line connects double-locus variants, and a dashed line connects STs separated by three or more allelic differences. Background shading highlights clonal complexes at a distance of two alleles in this dataset.

The minimum spanning tree of *C. jejuni* and *C. coli* STs was created from MLST allelic profiles using BIONUMERICS 8.0 (Applied Maths NV, Saint-Martens-Latem, Belgium). The tree shows 78 STs grouped into 12 previously characterized CCs, including both STs identified in this study (13) and 65 STs reported in the literature as predominant in *Campylobacter* strains of human and poultry origins. ST-460 and ST-10613, belonging to CC-460, are clustered together, and they share six of the seven HKGs, with the only difference being in *gln*A allele. The STs 6238, 607, and 3611 form another cluster belonging to the same CC-607 at the center of the tree. ST-51 belonging to CC-443 is located far from the other STs identified in this study. Of the three newly assigned STs, it can be concluded that ST-10645 is closely related to ST-8994, both belonging to CC-52, while ST-10647 is closely related to ST-3611, both belonging to CC-607. We also included STs that have been previously identified in *Campylobacter* strains isolated from poultry, human, and cattle fecal samples from the database (see text footnote 2). It can be concluded that CCs of this study are closely related to other CCs (257, 353, and 354) and distanced from CC-45 and CC-21 commonly reported in *Campylobacter* strains of poultry origin in Asia and elsewhere. The STs for *C. coli* from this study all belong to CC-1150 along with other STs obtained from the database (Figure 4).

## Discussion

Chicken contamination or infection by *Campylobacter* at the farm level usually affects the whole poultry production chain from farm to fork (Giannatale et al., 2019; Tang et al., 2020), and suitable interventions have to be adopted to reduce transmission from poultry to humans (Alter et al., 2011). The detection rate of *Campylobacter* was 35.9%, with *C. jejuni* being predominant (89.1%) compared to *C. coli* (10.9%). The detection rate was slightly higher compared to the rate previously reported for layers in the United States (Rama et al., 2018) but lower than the rates reported in the Netherlands (Schets et al., 2017) and Sri Lanka (Kalupahana et al., 2013). *C. jejuni* is reported to be the predominant species causing human campylobacteriosis, and our results concur with the literature (Han et al., 2007; Wei et al., 2014). However, exceptions have been reported, where *C. coli* was the predominant or the only isolated species (Marinou et al., 2012; Wieczorek et al., 2020a).

*Campylobacter* species exhibited high-level resistance to some antimicrobials (CIP, NAL, and TET), which concurs with findings previously reported in Korea (Kim et al., 2010; Lee et al., 2017; Oh et al., 2017) and elsewhere (Elhadidy et al., 2018; Zhang et al., 2020). In Korea, the use of ciprofloxacin was banned in 2010 (Ku et al., 2011), but mass medication of poultry with FQs, especially enrofloxacin, is still allowed (Seo and Lee, 2020). We characterized the *gyr*A gene, and the point mutation (C257T) confirmed the phenotypic results. The (C257T) mutation leads to increased resistance to FQs (Frasao et al., 2015) often used in livestock (Perrin-Guyomard et al., 2020) and in humans for treating undiagnosed diarrhea cases (Sproston et al., 2018). Surprisingly, quinolone-resistant *Campylobacter* strains have been reported in Australia in the absence of their use (Abraham et al., 2020). Although the use of FQs is banned in several countries, resistant strains are maintained in bacterial populations, which may explain their continued occurrence in humans and animals (Sproston et al., 2018). The association of using FQs in animal husbandry and the increased occurrence of AMR pathogens differ depending on the poultry production system, surveillance programs, and geographic location (Hao et al., 2016). It is hypothesized that the increased rates of human campylobacteriosis in Asia, Europe, and America are partly driven by a widespread prevalence of *C. jejuni* strains resistant to quinolones *via* poultry (Oh et al., 2017).

There was high resistance to TET, which concurs with previous reports in Korea (Wei et al., 2014; Lee et al., 2017), China (Zhang et al., 2020), and India (Kabir et al., 2015). TET is often used in poultry and pig industries based on their low cost and easy administration to animals through drinking water (Jonker and Picard, 2010). The sequencing and BLAST search showed the similarity of *tet*(O) gene sequences with known plasmid-mediated *tet*(O) gene sequences in the GenBank (Elhadidy et al., 2018; Lynch et al., 2020).

All isolates were susceptible to SIT (MIC values: 0.125–1 μg/ml) which possesses specific features lacking in other quinolones like a cyclopropyl ring with fluorine at R-1 and a chloride substituent at R-8 (Changkwanyeun et al., 2016), which may explain its high effectiveness. Our results corroborate previous reports with a MIC of 0.25 μg/ml (Yabe et al., 2010; Changkwanyeun et al., 2015). SIT is a candidate for clinical trials on campylobacteriosis (Changkwanyeun et al., 2015), and it is used for the eradication of multi-drug resistant *Helicobacter pylori* (Pohl et al., 2019). This could be a breakthrough as the alternative therapies for severe campylobacteriosis are very limited (Pavlova et al., 2020).

The low resistance to ERY and GEN shown by the isolates of this study has also been reported in different countries like Korea, Vietnam, and China (Carrique-Mas et al., 2014; Wei et al., 2014; Zhang et al., 2020). Resistance to ERY has been low and stable in China (Zhang et al., 2020), the United States, and across Europe (Tang et al., 2017). The limited resistance to ERY may be partly explained by a slower process of developing resistant strains when exposed to ERY and reduced survival of resistant strains (Luangtongkum et al., 2009, 2012). Resistance to GEN has also been relatively low as it is used for treating systemic infections (Lynch et al., 2020). Prudent use of existing antimicrobials and efforts to discover new alternative treatment options would help in curbing the AMR trend.

The *Campylobacter* species virulome contributes to their pathogenicity (Han et al., 2019). In this study, *cst*II, *cdt*B, *fla*A, *cad*F, and *dna*J were detected in all isolates. The detection rates were similar to a previous report in Korea (Oh et al., 2017) but higher than those reported in South Africa and Chile (González-Hein et al., 2013; Otigbu et al., 2018; Pillay et al., 2020). *cad*F seems to be a prerequisite for the invasion of epithelial cells by any bacterial pathogen (Ramires et al., 2020). The outer membrane phospholipase A (*pld*A) was observed more in *C. jejuni* than in *C. coli* (Figure 2), which corroborates the study in South Africa (Pillay et al., 2020), while for *cia*B, our findings are above those of a previous report in Korea (Oh et al., 2017) and contrast with the study in South Africa (Pillay et al., 2020). The presence of *cad*F and *cia*B facilitates the adhesion and internalization of *Campylobacter* in cellular models (Ramires et al., 2020). The detection rate for *csr*A in *C. jejuni* was slightly higher than the rate in *C. coli*, but *csr*A was lacking in *Campylobacter* strains from South Africa (Otigbu et al., 2018). None of our isolates expressed *ggt*. The latter was reported to be only 5.5% in Chile (González-Hein et al., 2013), but our findings contrast with the high values (30.9–43.2%) reported in Finland (Gonzalez et al., 2009). The difference could be associated with the complexity of the colonization process involving several genes and the use of strains from a single sampling site (González-Hein et al., 2013). Furthermore, MDR and virulent *C. jejuni* strains have been found more in summer than in winter, suggesting the role played by climate in the expression of some genes (Kim et al., 2019). We also evoke that several virulence genes are plasmid-mediated, which may affect their presence in different strains (Oh et al., 2017). The virulence genes reported in this study have been previously reported in *Campylobacter* strains isolated from humans (González-Hein et al., 2013; Oh et al., 2017), highlighting the potential virulence of these *Campylobacter* strains in causing human infections.

The *fla*A-RFLP typing showed a considerable diversity of *Campylobacter* strains despite being from the same farm. In our study, 26 types were found by *fla*A typing of 42 *Campylobacter* isolates. In another study in Korea, 30 *fla*A types were reported for 100 *C. jejuni* from chicken (Han et al., 2007), while 19 *fla*A types were reported for 100 *C. jejuni* from Grenada, Puerto Rico, and Alabama (Behringer et al., 2011). The *fla*A typing is suitable for laboratories dealing with a small number of isolates as it is cheaper and reproducible (Behringer et al., 2011). However, the drawback of the *flaA* typing is increased recombination events in the *flaA* gene, which modifies RFLP profiles (Harrington et al., 1997). Furthermore, the *fla*A typing focuses only on a single gene from a considerable genome, and there is a lack of inter-laboratory comparison of obtained results (Nguyen et al., 2016) due to the absence of *fla*A database. Therefore, a combination of several typing methods is recommended (Wieczorek and Osek, 2008).

Molecular typing techniques showed that 80% of human campylobacteriosis is associated with *Campylobacter* of poultry origin (Newell et al., 2011). In this study, the MLST revealed 10 STs for *C. jejuni* and three STs for *C. coli*. Three STs were new but could fit in existing clonal complexes. The predominant STs (3611 and 460) have been previously isolated in *C. jejuni* from poultry, while ST-51 was found in *C. jejuni* of both chicken and human origins in various regions of Korea (Oh et al., 2017). In Korea, in addition to the CCs identified in this study, other CCs including CC-48, CC-21, and CC-45 (Figure 4) have been recovered from *Campylobacter* isolates of poultry and human origins (Wei et al., 2014; Oh et al., 2017). The predominance of these clonal complexes and STs could be associated with environmental factors (geography and climate) and increasing poultry consumption in Korea (Oh et al., 2017). Globally, CC-45 has been found in various hosts, including poultry, cattle, dogs, wild birds, penguins, and it was also isolated from environmental samples (Cody et al., 2012; Shin et al., 2013), while CC-607 is largely associated with chicken and humans as reported from the United Kingdom, France, Canada, Thailand, and Uruguay (Cody et al., 2012; Duarte et al., 2014; Guyard-Nicodème et al., 2015). ST-45 and ST-50, predominant in Korea, are also common in *C. jejuni* from chickens in Europe (Wieczorek et al., 2020b). In 2019, ST-21, ST-50, and ST-137 have been isolated from wild ducks, indicating their widespread distribution (Wei et al., 2019). Three of the 10 STs (607, 443, and 51) were found in Japan and China (Ozawa et al., 2016; Ma et al., 2017), while CC-460 and CC-607 have also been reported in samples from humans and cattle, indicating their threat to public health (Ozawa et al., 2016; Kiatsomphob et al., 2019; Wei et al., 2019). CC-460 and CC-607 are thought to be virulent as they both possess many virulence genes. In Israel, type VI secretion system (T6SS), implicated in virulence, metabolism, AMR and contributing to host adaptation, has been found in both CCs (Rokney et al., 2018). In Japan, CC-21 is known as the prevalent clonal complex in human-derived *C. jejuni* isolates sharing a common genetic background and similar antimicrobial susceptibilities with *C. jejuni* strains from chicken (Ohishi et al., 2017). CC-21 (ST-50 and ST-21) could be of interest in the region as it has been found in Korea, Japan, and China from humans, poultry, and cattle (Ozawa et al., 2016; Oh et al., 2017; Kiatsomphob et al., 2019; Wei et al., 2019; Zhang et al., 2020). However, further studies are needed to confirm this hypothesis. Except for ST-257, other STs (ST-21, ST-48, and ST-353) identified in Korea have been recovered from *C. jejuni* of human origin in the United Kingdom, Brazil, and China (Gomes et al., 2019). It is known that the predominance of certain genotypes of *C. jejuni* depends on several factors such as animal reservoirs, zoonotic transmission, rates of recombination events, food source, and the first time when a given genotype is recorded in the country (Rokney et al., 2018).

Although resistance to quinolones and TET was not a particularity of certain STs, a strong correlation between CC-460, CC-607, CC-45, CC-48, CC-21, and resistance to both TET and quinolones in *C. jejuni* strains is not new (Cody et al., 2012; Shin et al., 2013; Guyard-Nicodème et al., 2015; Zhang et al., 2020). In Korea, *C. jejuni* strains of human origin with ST-607, ST-137, ST-45, ST-21, and ST-48 were found to be MDR (Shin et al., 2013). Two isolates of *C. jejuni* belonged to CC-443, which is suggested to harbor antibiotic-resistant and pathogenic *C. jejuni* strains (Kim et al., 2019). There is limited literature associating virulence genes to specific CCs and STs; thus, this study highlights the virulence potential of the presented *Campylobacter* isolates. For *C. coli*, ST-1121 has been reported from broilers in China (Tang et al., 2020). We suggest carrying out WGS to have a detailed picture of the pathogenicity by analyzing all the housekeeping, virulence, and AMR genes. The WGS would also enlighten the evolutionary pathways of the *Campylobacter* spp. used in this study to inform better practices that may lead to a reduction of campylobacteriosis cases.

The limitation of this study was the restricted access to various poultry farms across the city which would have given a broad picture of the prevalence, AMR profiles, and genotypes associated with *Campylobacter* in Gangneung. *Campylobacter* strains from the current study proved to be highly diverse considering the number of obtained STs and CCs. This is in agreement with other published studies reporting the possibility of getting several genotypes in a single poultry flock, suggesting different exposure sources *via* horizontal transmission and/or genetic drifts within the *Campylobacter* population (Alter et al., 2011; El-Adawy et al., 2013).

## Conclusion

This study highlights the role of layers as a reservoir of *Campylobacter* spp., harboring various AMR and virulence genes. The genotyping highlighted that *C. jejuni* isolates were more diverse than *C. coli* as analyzed by MLST. The MLST revealed that CC-607 (ST-3611) and CC-460 (ST-460) were the predominant ones, while three STs were newly assigned. ERY, GEN, and SIT need to be appropriately used to prevent or delay the increasing resistance in *Campylobacter* species. The isolates of this study may present a potential hazard to public health based on their AMR profiles, virulence genes, and genotyping data.

## Data Availability Statement

The datasets presented in this study can be found in online repositories. The names of the repository/repositories and accession number(s) can be found in the article/Supplementary Material.

## Ethics Statement

Ethical review and approval was not required for the animal study.

## Author Contributions

NG, C-HP, and EK conceived the study. NG carried out the experiments, analyzed and interpreted the data, and wrote the manuscript. D-GS and K-YY substantially contributed to the analysis of the results. LM, MM, DM, and RA substantially revised the manuscript. All authors read and approved the final version of the manuscript.

## Conflict of Interest

The authors declare that the research was conducted in the absence of any commercial or financial relationships that could be construed as a potential conflict of interest.

## Footnotes

1 http://www.eucast.org/

2 http://pubmlst.org/campylobacter
