# Supplementary figures and images for: Antimicrobial Resistance Profiles, Virulence Genes, and Genetic Diversity of Thermophilic Campylobacter Species Isolated From a Layer Poultry Farm in Korea
Source: Front Microbiol. 2021 Mar 29;12:622275. doi: 10.3389/fmicb.2021.622275 (PMC8043113; doi:10.3389/fmicb.2021.622275)

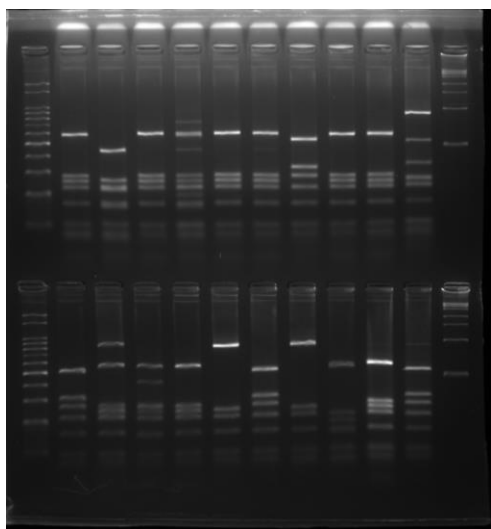

Band for run 1 and 2

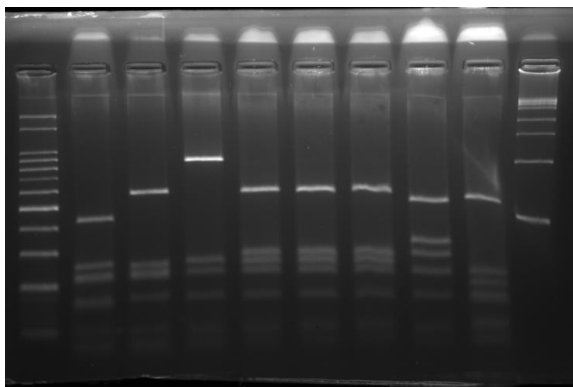

Band for run 3

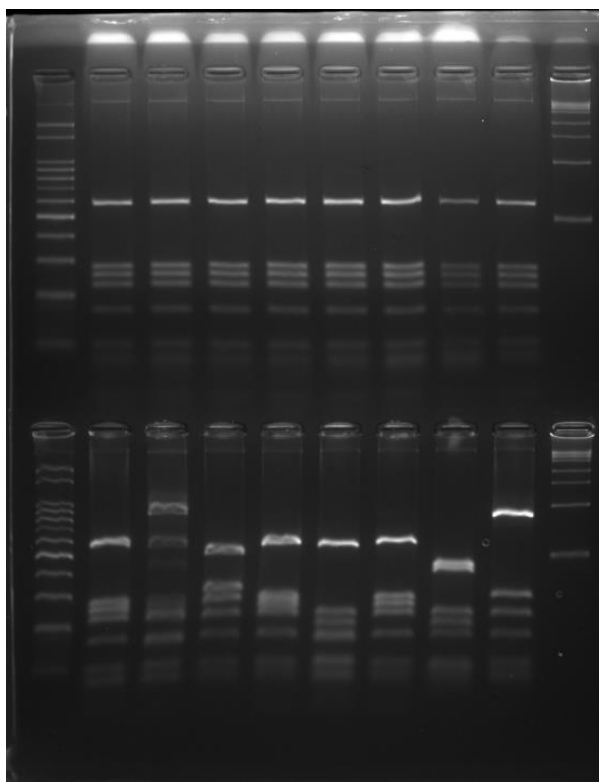

Band for run 4 and 5

Supplement: Supplementary file 1 [file Data_Sheet_1.pdf]
