# Supplementary material for: Antimicrobial Resistance Profiles, Virulence Genes, and Genetic Diversity of Thermophilic Campylobacter Species Isolated From a Layer Poultry Farm in Korea
Source: Front Microbiol. 2021 Mar 29;12:622275. doi: 10.3389/fmicb.2021.622275 (PMC8043113; doi:10.3389/fmicb.2021.622275)
Supplement: Supplementary file 2 [file Data_Sheet_2.PDF]

Table 1: Primers used for species, antimicrobial resistance, and virulence genes confirmation

Target gene	Direction	Sequence (5' -3')	Amplicon size	Annealing temperature (ºC)	Reference
16S rRNA	Forward	GGATGACACTTTTCGGAGC	816	55	(Yamazaki-Matsune et al., 2007)
	Reverse	CATTGTAGCACGTGTGTC			
cj0414	Forward	CAAATAAAGTTAGAGGTAGAATGT	161		
	Reverse	CCATAAGCACTAGCTAGCTGAT			
ask	Forward	GGTATGATTTCTACAAAGCGAG	502		
	Reverse	ATAAAAGACTATCGTCGCGTG			
tet(O)	Forward	GCGTTTTGTTTATGTGCG	559	55	(Price et al., 2005; Obeng et al., 2012)
	Reverse	ATGGACAACCCGACAGAAG			
*cjgyr*A	Forward	GCCTGACGCAAGAGATGGTTTA	454		
	Reverse	TATGAGGCGGGATGTTTGTCG			
*cme*B	Forward	TCCTAGCAGCACAATATG	241		
	Reverse	AGCTTCGATAGCTGCATC			
*cst*II	Forward	CAG CTT TCT ATT GCC CTT GC	570	52	(Otigbu et al., 2018)
	Reverse	ACACATATAGACCCCTGAGG			
*cdt*B	Forward	CACGGTTAAAATCCCCTGCT	495		
	Reverse	GCACTTGGAATTTGCAAGGC			
*fla*A	Forward	GGATTTCGTATTAACACAAATGGTGC	1725	48	(Wieczorek & Osek, 2008)
	Reverse	CTGTAGTAATCTTAAAACATTTTG			
ggt	Forward	GAG TGC TAT GCT TGA TCG CT	419	58	(González-Hein et al., 2013)
	Reverse	TAG GTG GCG ACA TGG AAA TG			
csrA	Forward	CAC AGT CAG TGA AGG TGC TT	878	52	(Otigbu et al., 2018)
	Reverse	ACT CGC ACA ATC GCT ACT TC			
*cad*F	Forward	TTGAAGGTAATTTAGATATG	400	42	(Otigbu et al., 2018)
	Reverse	CTAATACCTAAAGTTGAAAC			
*cia*B	Forward	TGCGAGATTTTTCGAGAATG	527	54	(Chansiripornchai & Sasipreeyajan, 2009)
	Reverse	TGCCCGCCTTAGAACTTACA			
*pld*A	Forward	AAGAGTGAGGCGAAATTCCA	385	46	(Chansiripornchai & Sasipreeyajan, 2009)
	Reverse	GCAAGATGGCAGGATTATCA			
*dna*J	Forward	ATTGATTTTGCTGCGGGTAG	177	50	(Chansiripornchai & Sasipreeyajan, 2009)
	Reverse	ATCCGCAAAAGCTTCAAAAA			
